# Supplementary material for: Blood glucose, insulin and glycogen profiles in Sprague-Dawley rats co-infected with Plasmodium berghei ANKA and Trichinella zimbabwensis
Source: PeerJ. 2022 Jul 29;10:e13713. doi: 10.7717/peerj.13713 (PMC9341445; doi:10.7717/peerj.13713)

**BLOOD GLUCOSE CONCENTRATION STATISTICS**

**GLUCOSE TWO-WAY ANOVA**

| **Table Analyzed** | **BLOOD GLUCOSE PAPER GRAPH** | | |  |  |
| --- | --- | --- | --- | --- | --- |
|  |  |  |  |  |  |
| Two-way ANOVA | |  |  |  |  |
|  |  |  |  |  |  |
| Source of Variation | % of total variation | P value |  |  |  |
| Interaction | 13.1 | 0.0012 |  |  |  |
| Treatment | 12.36 | 0.0001 |  |  |  |
| Day post infection | 7.99 | 0.0011 |  |  |  |
|  |  |  |  |  |  |
| Source of Variation | P value summary | Significant? |  |  |  |
| Interaction | ** | Yes |  |  |  |
| Treatment | *** | Yes |  |  |  |
| Day post infection | ** | Yes |  |  |  |
|  |  |  |  |  |  |
| Source of Variation | Df | Sum-of-squares | Mean square | F |  |
| Interaction | 6 | 23.47 | 3.911 | 3.95 |  |
| Treatment | 3 | 22.14 | 7.381 | 7.454 |  |
| Day post infection | 2 | 14.31 | 7.156 | 7.227 |  |
| Residual | 132 | 130.7 | 0.9902 |  |  |
|  |  |  |  |  |  |
| Number of missing values | 72 |  |  |  |  |
|  |  |  |  |  |  |
| Bonferroni posttests | |  |  |  |  |
|  |  |  |  |  |  |
| Control vs Malaria | |  |  |  |  |
| Day post infection | Control | Malaria | Difference | 95% CI of diff. |  |
| 0.0000 | 5.728 | 5.783 | 0.05555 | -0.9557 to 1.067 |  |
| 7.000 | 6.183 | 4.958 | -1.225 | -2.463 to 0.01349 | |
| 14.00 | 5.367 | 3.083 | -2.283 | -4.035 to -0.5318 | |
|  |  |  |  |  |  |
| Day post infection | Difference | t | P value | Summary |  |
| 0.0000 | 0.05555 | 0.1675 | P > 0.05 | ns |  |
| 7.000 | -1.225 | 3.015 | P<0.01 | ** |  |
| 14.00 | -2.283 | 3.974 | P<0.001 | *** |  |
|  |  |  |  |  |  |
| Control vs Trichinella | |  |  |  |  |
| Day post infection | Control | Trichinella | Difference | 95% CI of diff. |  |
| 0.0000 | 5.728 | 5.717 | -0.01111 | -1.022 to 1.000 |  |
| 7.000 | 6.183 | 5.25 | -0.9333 | -2.172 to 0.3052 | |
| 14.00 | 5.367 | 5.417 | 0.05 | -1.701 to 1.801 |  |
|  |  |  |  |  |  |
| Day post infection | Difference | t | P value | Summary |  |
| 0.0000 | -0.01111 | 0.0335 | P > 0.05 | ns |  |
| 7.000 | -0.9333 | 2.297 | P > 0.05 | ns |  |
| 14.00 | 0.05 | 0.08703 | P > 0.05 | ns |  |
|  |  |  |  |  |  |
| Control vs Malaria + Trichinella | |  |  |  |  |
| Day post infection | Control | Malaria + Trichinella | Difference | 95% CI of diff. |  |
| 0.0000 | 5.728 | 5.589 | -0.1389 | -1.150 to 0.8723 |  |
| 7.000 | 6.183 | 5.533 | -0.65 | -1.888 to 0.5885 | |
| 14.00 | 5.367 | 5.383 | 0.01667 | -1.735 to 1.768 |  |
|  |  |  |  |  |  |
| Day post infection | Difference | t | P value | Summary |  |
| 0.0000 | -0.1389 | 0.4187 | P > 0.05 | ns |  |
| 7.000 | -0.65 | 1.6 | P > 0.05 | ns |  |
| 14.00 | 0.01667 | 0.02901 | P > 0.05 | ns |  |
|  |  |  |  |  |  |
| Malaria vs Trichinella | |  |  |  |  |
| Day post infection | Malaria | Trichinella | Difference | 95% CI of diff. |  |
| 0.0000 | 5.783 | 5.717 | -0.06667 | -1.078 to 0.9446 | |
| 7.000 | 4.958 | 5.25 | 0.2917 | -0.9468 to 1.530 | |
| 14.00 | 3.083 | 5.417 | 2.333 | 0.5818 to 4.085 |  |
|  |  |  |  |  |  |
| Day post infection | Difference | t | P value | Summary |  |
| 0.0000 | -0.06667 | 0.201 | P > 0.05 | ns |  |
| 7.000 | 0.2917 | 0.718 | P > 0.05 | ns |  |
| 14.00 | 2.333 | 4.061 | P<0.001 | *** |  |
|  |  |  |  |  |  |
| Malaria vs Malaria + Trichinella | |  |  |  |  |
| Day post infection | Malaria | Malaria + Trichinella | Difference | 95% CI of diff. |  |
| 0.0000 | 5.783 | 5.589 | -0.1944 | -1.206 to 0.8168 | |
| 7.000 | 4.958 | 5.533 | 0.575 | -0.6635 to 1.813 |  |
| 14.00 | 3.083 | 5.383 | 2.3 | 0.5485 to 4.051 |  |
|  |  |  |  |  |  |
| Day post infection | Difference | t | P value | Summary |  |
| 0.0000 | -0.1944 | 0.5862 | P > 0.05 | ns |  |
| 7.000 | 0.575 | 1.415 | P > 0.05 | ns |  |
| 14.00 | 2.3 | 4.003 | P<0.001 | *** |  |
|  |  |  |  |  |  |
| Trichinella vs Malaria + Trichinella | |  |  |  |  |
| Day post infection | Trichinella | Malaria + Trichinella | Difference | 95% CI of diff. |  |
| 0.0000 | 5.717 | 5.589 | -0.1278 | -1.139 to 0.8834 | |
| 7.000 | 5.25 | 5.533 | 0.2833 | -0.9552 to 1.522 |  |
| 14.00 | 5.417 | 5.383 | -0.03333 | -1.785 to 1.718 |  |
|  |  |  |  |  |  |
| Day post infection | Difference | t | P value | Summary |  |
| 0.0000 | -0.1278 | 0.3852 | P > 0.05 | ns |  |
| 7.000 | 0.2833 | 0.6975 | P > 0.05 | ns |  |
| 14.00 | -0.03333 | 0.05802 | P > 0.05 | ns |  |

**GLUCOSE NARRATIVE RESULTS**


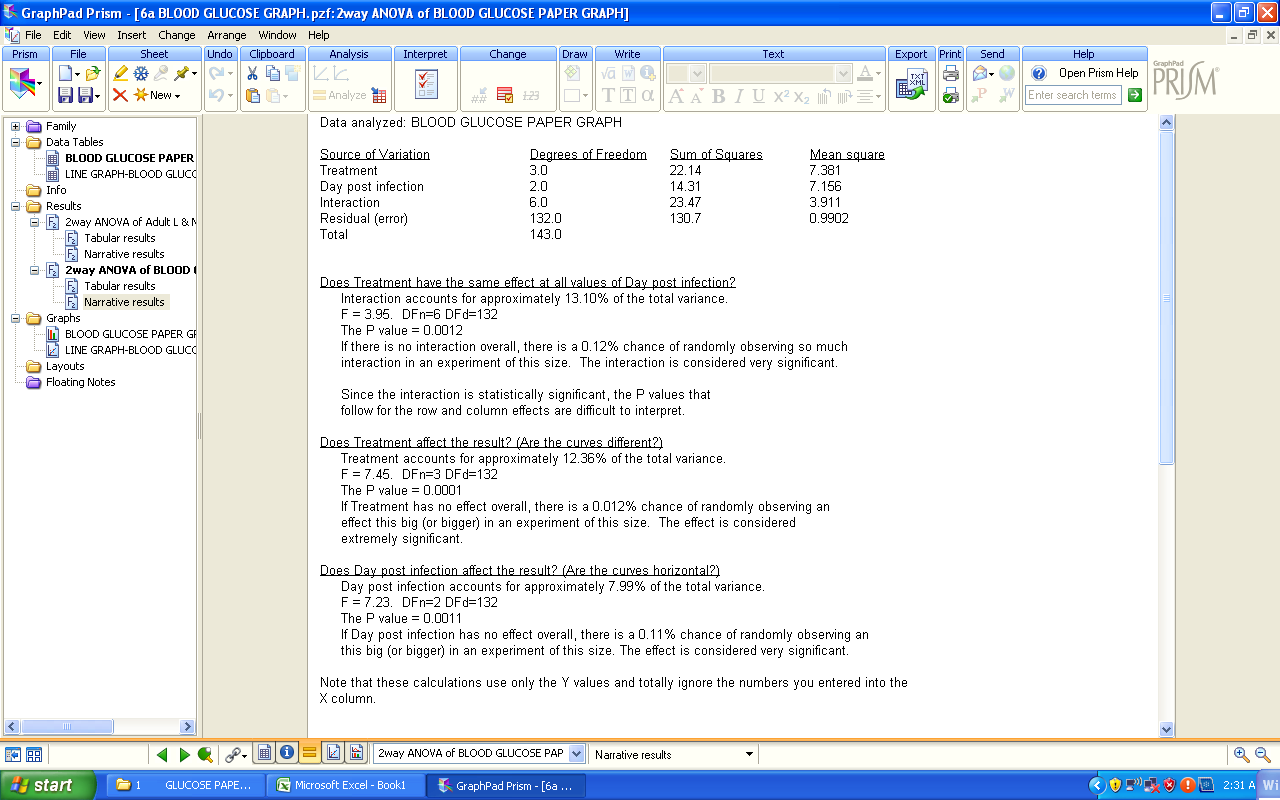


**INSULIN CONCENTRATION STATISTICS**

**INSULIN CONCENTRATION 2 WAY ANOVA**

| Table Analyzed | INSULIN RAW DATA-ANALYSIS ONLY | | |  |  |
| --- | --- | --- | --- | --- | --- |
|  |  |  |  |  |  |
| Two-way ANOVA | |  |  |  |  |
|  |  |  |  |  |  |
| Source of Variation | % of total variation | P value |  |  |  |
| Interaction | 11.72 | 0.1251 |  |  |  |
| Treatment | 19.23 | 0.0016 |  |  |  |
| Days | 2.05 | 0.4047 |  |  |  |
|  |  |  |  |  |  |
| Source of Variation | P value summary | Significant? |  |  |  |
| Interaction | ns | No |  |  |  |
| Treatment | ** | Yes |  |  |  |
| Days | ns | No |  |  |  |
|  |  |  |  |  |  |
| Source of Variation | Df | Sum-of-squares | Mean square | F |  |
| Interaction | 6 | 3129 | 521.5 | 1.75 |  |
| Treatment | 3 | 5134 | 1711 | 5.742 |  |
| Days | 2 | 547.4 | 273.7 | 0.9183 |  |
| Residual | 60 | 17880 | 298.1 |  |  |
|  |  |  |  |  |  |
| Number of missing values | 0 |  |  |  |  |
|  |  |  |  |  |  |
| Bonferroni posttests | |  |  |  |  |
|  |  |  |  |  |  |
| Control vs Malaria | |  |  |  |  |
| Days | Control | Malaria | Difference | 95% CI of diff. |  |
| 0.0000 | 32.62 | 32.62 | 0 | -31.10 to 31.10 |  |
| 7.000 | 32.62 | 24.56 | -8.061 | -39.16 to 23.04 |  |
| 14.00 | 32.58 | 16.59 | -15.99 | -47.09 to 15.11 |  |
|  |  |  |  |  |  |
| Days | Difference | t | P value | Summary |  |
| 0.0000 | 0 | 0 | P > 0.05 | ns |  |
| 7.000 | -8.061 | 0.8087 | P > 0.05 | ns |  |
| 14.00 | -15.99 | 1.604 | P > 0.05 | ns |  |
|  |  |  |  |  |  |
| Control vs Trichinella | |  |  |  |  |
| Days | Control | Trichinella | Difference | 95% CI of diff. |  |
| 0.0000 | 32.62 | 3.478 | -29.15 | -60.24 to 1.955 |  |
| 7.000 | 32.62 | 15.83 | -16.79 | -47.89 to 14.31 |  |
| 14.00 | 32.58 | 29.12 | -3.465 | -34.56 to 27.64 |  |
|  |  |  |  |  |  |
| Days | Difference | t | P value | Summary |  |
| 0.0000 | -29.15 | 2.924 | P < 0.05 | * |  |
| 7.000 | -16.79 | 1.685 | P > 0.05 | ns |  |
| 14.00 | -3.465 | 0.3476 | P > 0.05 | ns |  |
|  |  |  |  |  |  |
| Control vs Malaria + Trichinella | |  |  |  |  |
| Days | Control | Malaria + Trichinella | Difference | 95% CI of diff. |  |
| 0.0000 | 32.62 | 3.478 | -29.15 | -60.24 to 1.955 |  |
| 7.000 | 32.62 | 7.221 | -25.4 | -56.50 to 5.698 |  |
| 14.00 | 32.58 | 20.27 | -12.31 | -43.41 to 18.79 |  |
|  |  |  |  |  |  |
| Days | Difference | t | P value | Summary |  |
| 0.0000 | -29.15 | 2.924 | P < 0.05 | * |  |
| 7.000 | -25.4 | 2.549 | P < 0.05 | * |  |
| 14.00 | -12.31 | 1.235 | P > 0.05 | ns |  |
|  |  |  |  |  |  |
| Malaria vs Trichinella | |  |  |  |  |
| Days | Malaria | Trichinella | Difference | 95% CI of diff. |  |
| 0.0000 | 32.62 | 3.478 | -29.15 | -60.24 to 1.955 |  |
| 7.000 | 24.56 | 15.83 | -8.73 | -39.83 to 22.37 |  |
| 14.00 | 16.59 | 29.12 | 12.53 | -18.57 to 43.63 |  |
|  |  |  |  |  |  |
| Days | Difference | t | P value | Summary |  |
| 0.0000 | -29.15 | 2.924 | P < 0.05 | * |  |
| 7.000 | -8.73 | 0.8758 | P > 0.05 | ns |  |
| 14.00 | 12.53 | 1.257 | P > 0.05 | ns |  |
|  |  |  |  |  |  |
| Malaria vs Malaria + Trichinella | |  |  |  |  |
| Days | Malaria | Malaria + Trichinella | Difference | 95% CI of diff. |  |
| 0.0000 | 32.62 | 3.478 | -29.15 | -60.24 to 1.955 |  |
| 7.000 | 24.56 | 7.221 | -17.34 | -48.44 to 13.76 |  |
| 14.00 | 16.59 | 20.27 | 3.68 | -27.42 to 34.78 |  |
|  |  |  |  |  |  |
| Days | Difference | t | P value | Summary |  |
| 0.0000 | -29.15 | 2.924 | P < 0.05 | * |  |
| 7.000 | -17.34 | 1.74 | P > 0.05 | ns |  |
| 14.00 | 3.68 | 0.3692 | P > 0.05 | ns |  |
|  |  |  |  |  |  |
| Trichinella vs Malaria + Trichinella | |  |  |  |  |
| Days | Trichinella | Malaria + Trichinella | Difference | 95% CI of diff. |  |
| 0.0000 | 3.478 | 3.478 | 0 | -31.10 to 31.10 |  |
| 7.000 | 15.83 | 7.221 | -8.612 | -39.71 to 22.49 |  |
| 14.00 | 29.12 | 20.27 | -8.847 | -39.95 to 22.25 |  |
|  |  |  |  |  |  |
| Days | Difference | t | P value | Summary |  |
| 0.0000 | 0 | 0 | P > 0.05 | ns |  |
| 7.000 | -8.612 | 0.864 | P > 0.05 | ns |  |
| 14.00 | -8.847 | 0.8876 | P > 0.05 | ns |  |
|  |  |  |  |  |  |

**INSULIN CONCENTRATION NARRATIVE RESULTS**


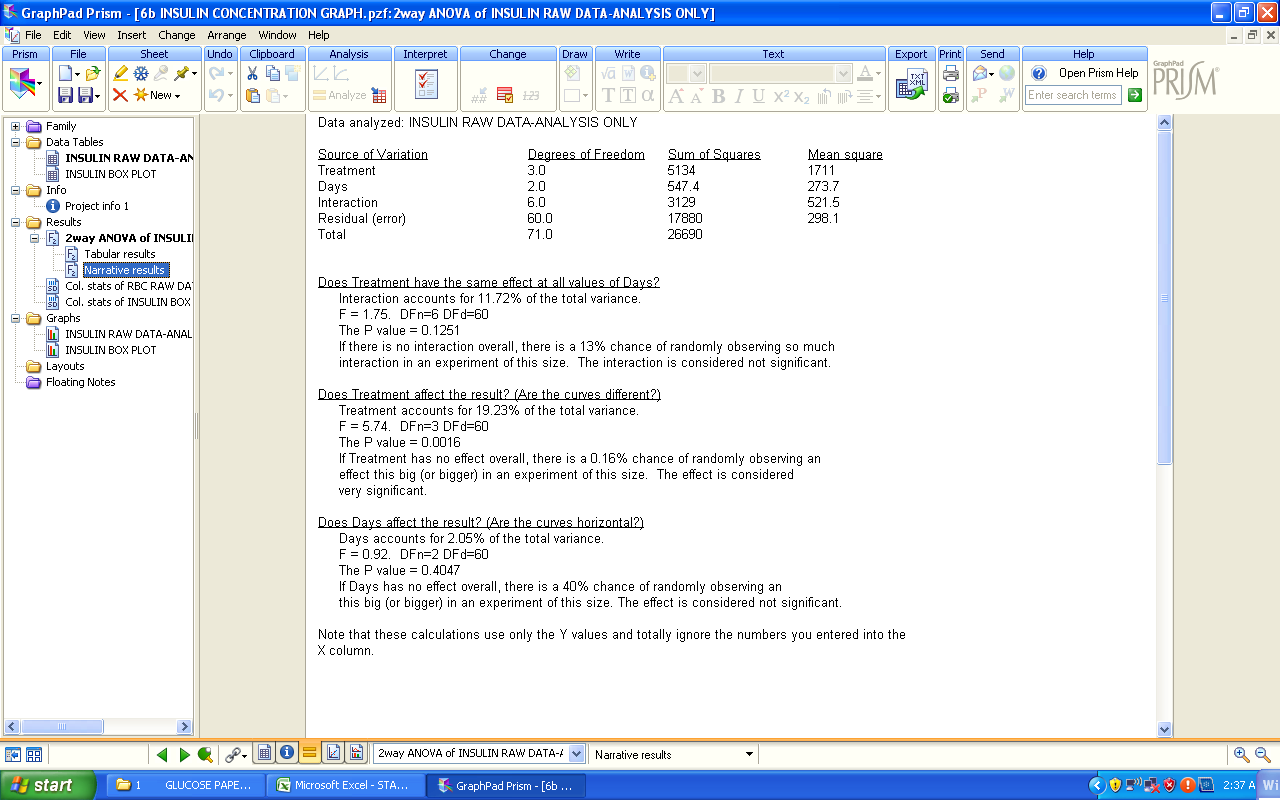


**INSULIN CONCENTRATION BOX PLOT STATISTICS**


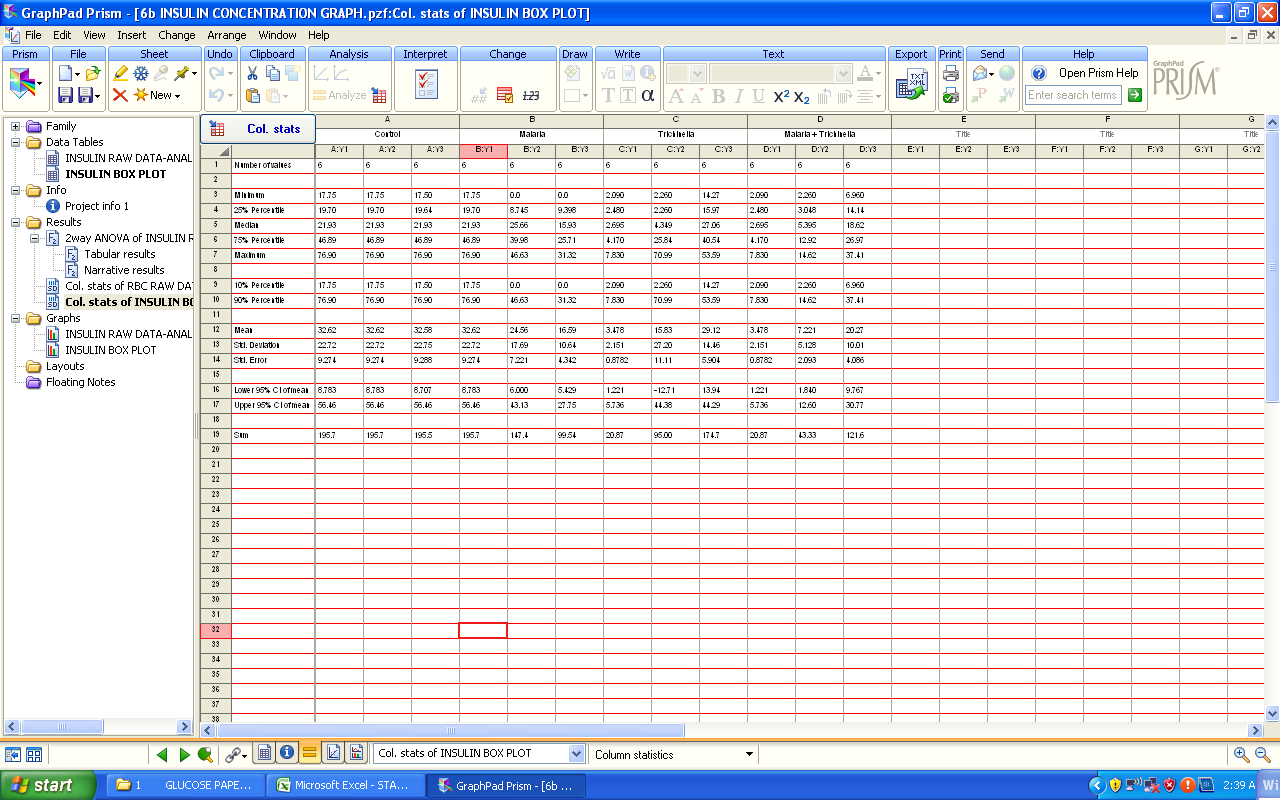


**LIVER GLYCOGEN CONCENTRATION STATISTICS**

**LIVER GLYCOGEN CONCENTRATION 2 WAY ANOVA**

| Table Analyzed | LIVER GLYCOGEN RAW DATA-ANALYSIS ONLY | | |  |  |
| --- | --- | --- | --- | --- | --- |
|  |  |  |  |  |  |
| Two-way ANOVA | |  |  |  |  |
|  |  |  |  |  |  |
| Source of Variation | % of total variation | P value |  |  |  |
| Interaction | 15.23 | 0.01 |  |  |  |
| Treatment | 20.53 | P<0.0001 |  |  |  |
| Days | 15.44 | 0.0003 |  |  |  |
|  |  |  |  |  |  |
| Source of Variation | P value summary | Significant? |  |  |  |
| Interaction | ** | Yes |  |  |  |
| Treatment | *** | Yes |  |  |  |
| Days | *** | Yes |  |  |  |
|  |  |  |  |  |  |
| Source of Variation | Df | Sum-of-squares | Mean square | F |  |
| Interaction | 6 | 0.3753 | 0.06255 | 3.121 |  |
| Treatment | 3 | 0.5058 | 0.1686 | 8.413 |  |
| Days | 2 | 0.3805 | 0.1903 | 9.494 |  |
| Residual | 60 | 1.202 | 0.02004 |  |  |
|  |  |  |  |  |  |
| Number of missing values | 0 |  |  |  |  |
|  |  |  |  |  |  |
| Bonferroni posttests | |  |  |  |  |
|  |  |  |  |  |  |
| Control vs Malaria | |  |  |  |  |
| Days | Control | Malaria | Difference | 95% CI of diff. |  |
| 0.0000 | 0.6742 | 0.6497 | -0.0245 | -0.2795 to 0.2305 | |
| 7.000 | 0.6365 | 0.6232 | -0.01333 | -0.2684 to 0.2417 | |
| 14.00 | 0.6163 | 0.6115 | -0.004833 | -0.2599 to 0.2502 | |
|  |  |  |  |  |  |
| Days | Difference | t | P value | Summary |  |
| 0.0000 | -0.0245 | 0.2998 | P > 0.05 | ns |  |
| 7.000 | -0.01333 | 0.1631 | P > 0.05 | ns |  |
| 14.00 | -0.004833 | 0.05914 | P > 0.05 | ns |  |
|  |  |  |  |  |  |
| Control vs Trichinella | |  |  |  |  |
| Days | Control | Trichinella | Difference | 95% CI of diff. |  |
| 0.0000 | 0.6742 | 0.6207 | -0.0535 | -0.3085 to 0.2015 | |
| 7.000 | 0.6365 | 0.2663 | -0.3702 | -0.6252 to -0.1151 | |
| 14.00 | 0.6163 | 0.5625 | -0.05383 | -0.3089 to 0.2012 | |
|  |  |  |  |  |  |
| Days | Difference | t | P value | Summary |  |
| 0.0000 | -0.0535 | 0.6546 | P > 0.05 | ns |  |
| 7.000 | -0.3702 | 4.529 | P<0.001 | *** |  |
| 14.00 | -0.05383 | 0.6587 | P > 0.05 | ns |  |
|  |  |  |  |  |  |
| Control vs Malaria + Trichinella | |  |  |  |  |
| Days | Control | Malaria + Trichinella | Difference | 95% CI of diff. |  |
| 0.0000 | 0.6742 | 0.6392 | -0.035 | -0.2900 to 0.2200 | |
| 7.000 | 0.6365 | 0.349 | -0.2875 | -0.5425 to -0.03248 | |
| 14.00 | 0.6163 | 0.3768 | -0.2395 | -0.4945 to 0.01552 | |
|  |  |  |  |  |  |
| Days | Difference | t | P value | Summary |  |
| 0.0000 | -0.035 | 0.4282 | P > 0.05 | ns |  |
| 7.000 | -0.2875 | 3.518 | P<0.01 | ** |  |
| 14.00 | -0.2395 | 2.93 | P < 0.05 | * |  |
|  |  |  |  |  |  |
| Malaria vs Trichinella | |  |  |  |  |
| Days | Malaria | Trichinella | Difference | 95% CI of diff. |  |
| 0.0000 | 0.6497 | 0.6207 | -0.029 | -0.2840 to 0.2260 | |
| 7.000 | 0.6232 | 0.2663 | -0.3568 | -0.6119 to -0.1018 | |
| 14.00 | 0.6115 | 0.5625 | -0.049 | -0.3040 to 0.2060 | |
|  |  |  |  |  |  |
| Days | Difference | t | P value | Summary |  |
| 0.0000 | -0.029 | 0.3548 | P > 0.05 | ns |  |
| 7.000 | -0.3568 | 4.366 | P<0.001 | *** |  |
| 14.00 | -0.049 | 0.5995 | P > 0.05 | ns |  |
|  |  |  |  |  |  |
| Malaria vs Malaria + Trichinella | |  |  |  |  |
| Days | Malaria | Malaria + Trichinella | Difference | 95% CI of diff. |  |
| 0.0000 | 0.6497 | 0.6392 | -0.0105 | -0.2655 to 0.2445 | |
| 7.000 | 0.6232 | 0.349 | -0.2742 | -0.5292 to -0.01915 | |
| 14.00 | 0.6115 | 0.3768 | -0.2347 | -0.4897 to 0.02035 | |
|  |  |  |  |  |  |
| Days | Difference | t | P value | Summary |  |
| 0.0000 | -0.0105 | 0.1285 | P > 0.05 | ns |  |
| 7.000 | -0.2742 | 3.354 | P<0.01 | ** |  |
| 14.00 | -0.2347 | 2.871 | P < 0.05 | * |  |
|  |  |  |  |  |  |
| Trichinella vs Malaria + Trichinella | |  |  |  |  |
| Days | Trichinella | Malaria + Trichinella | Difference | 95% CI of diff. |  |
| 0.0000 | 0.6207 | 0.6392 | 0.0185 | -0.2365 to 0.2735 | |
| 7.000 | 0.2663 | 0.349 | 0.08267 | -0.1724 to 0.3377 | |
| 14.00 | 0.5625 | 0.3768 | -0.1857 | -0.4407 to 0.06935 | |
|  |  |  |  |  |  |
| Days | Difference | t | P value | Summary |  |
| 0.0000 | 0.0185 | 0.2263 | P > 0.05 | ns |  |
| 7.000 | 0.08267 | 1.011 | P > 0.05 | ns |  |
| 14.00 | -0.1857 | 2.272 | P > 0.05 | ns |  |

**LIVER GLYCOGEN CONCENTRATION NARRATIVE STATISTICS**


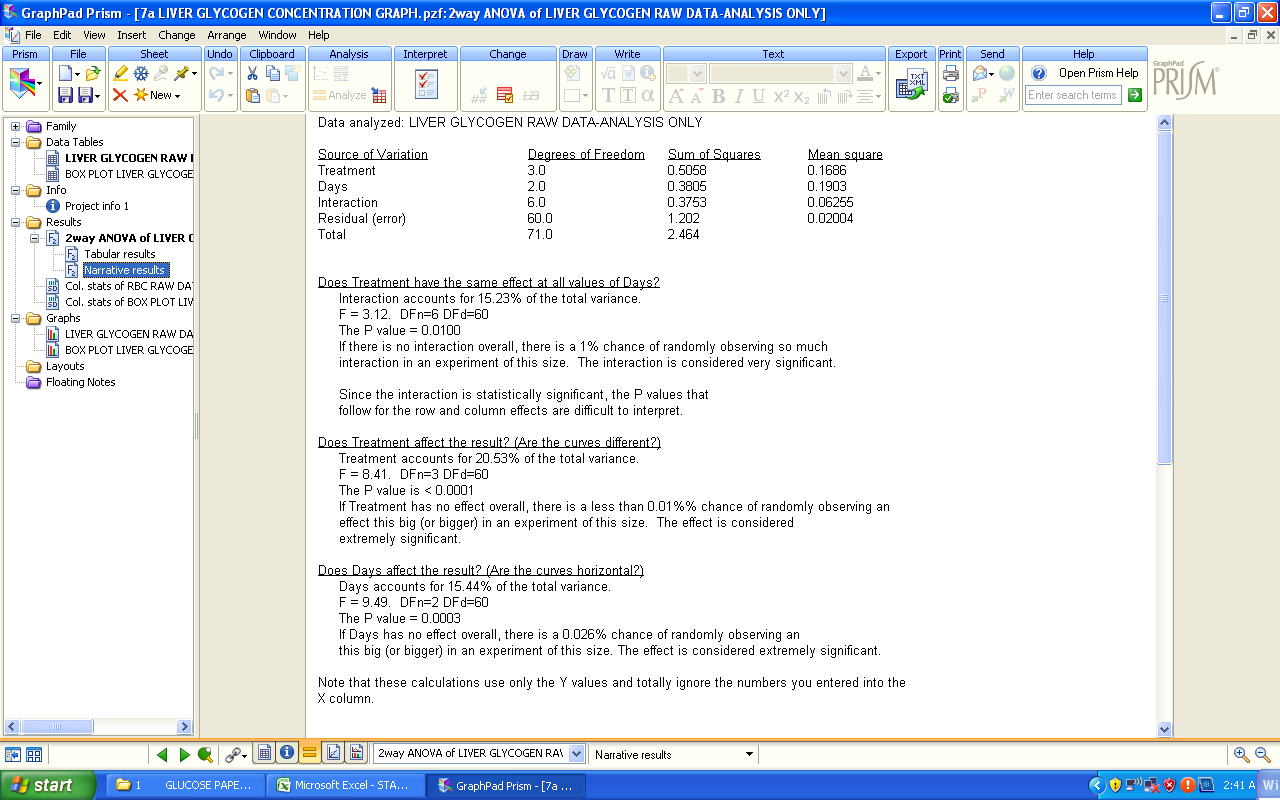


**LIVER GLYCOGEN CONCENTRATION BOX PLOT STATISTICS**


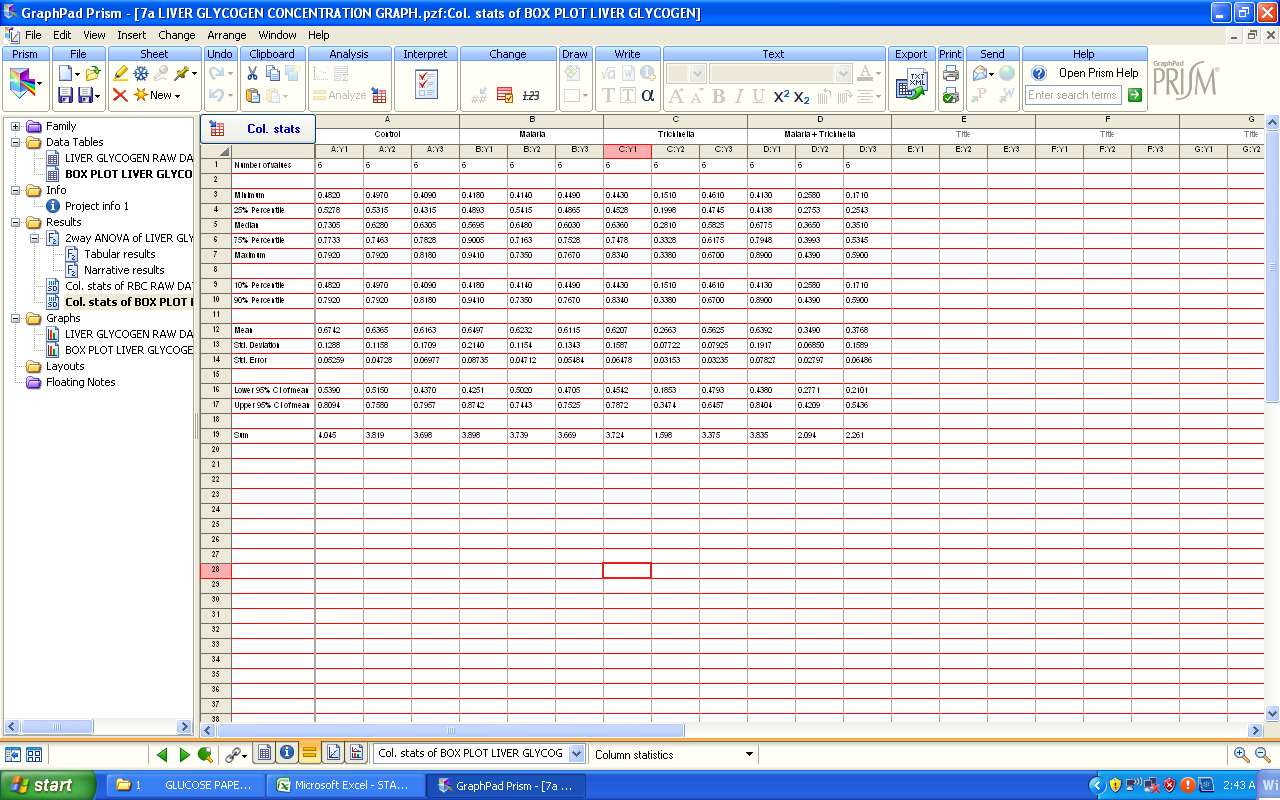


**MUSCLE GLYCOGEN CONCENTRATION STATISTICS**

**MUSCLE GLYCOGEN CONCENTRATION 2 WAY ANOVA**


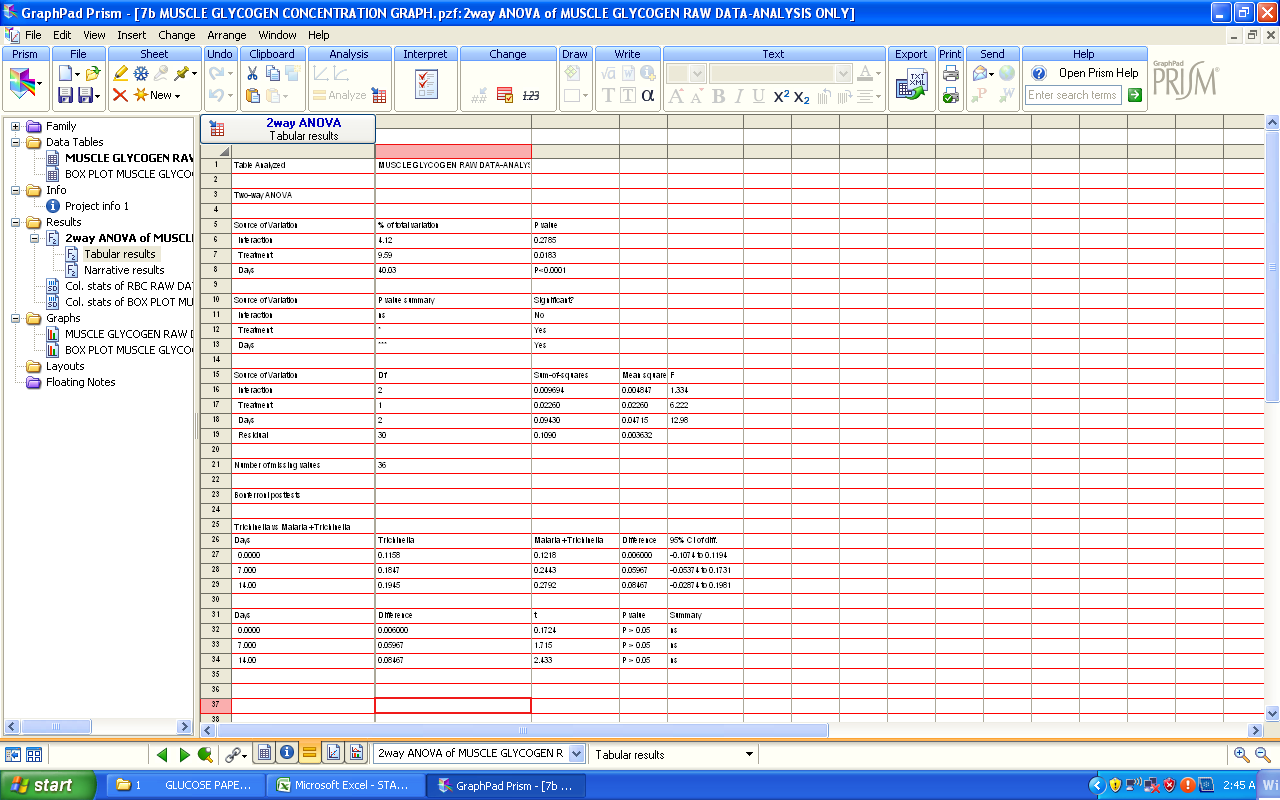


**MUSCLE GLYCOGEN CONCENTRATION NARRATIVE STATISTICS**


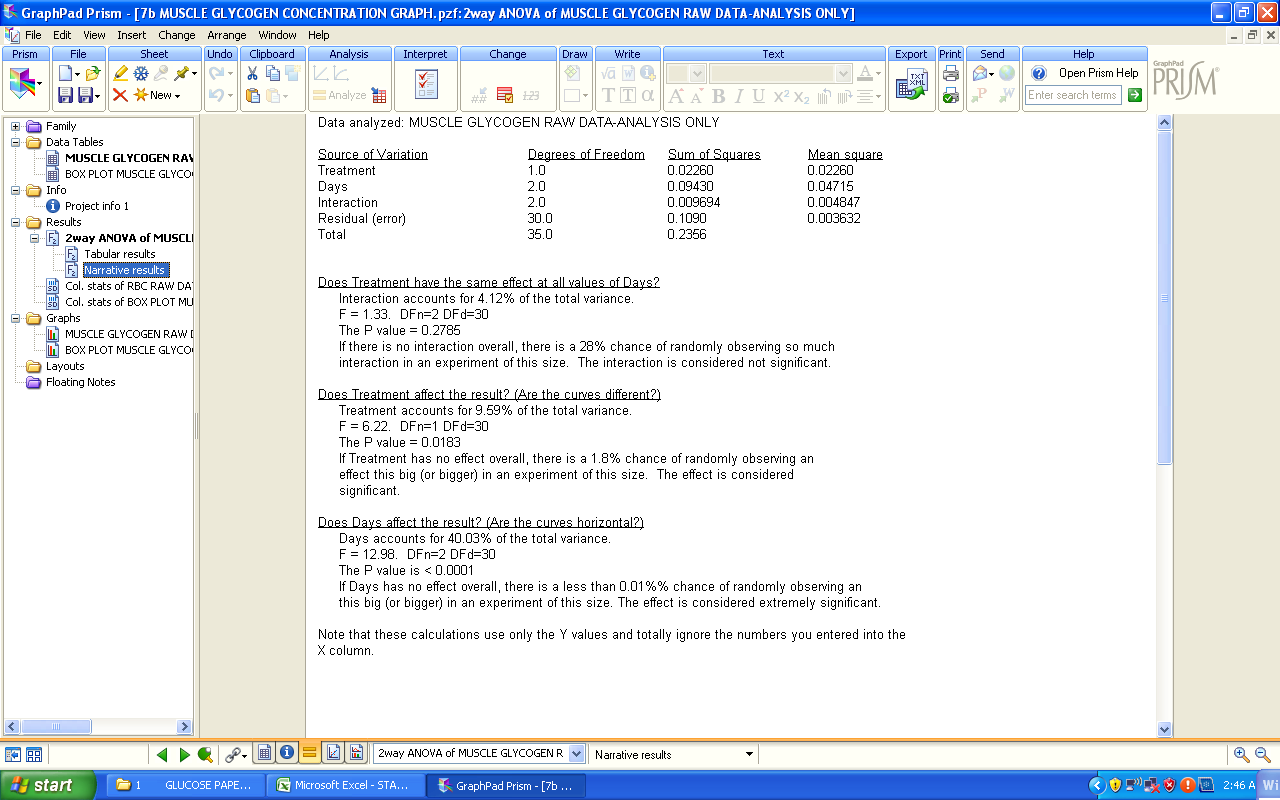


**MUSCLE GLYCOGEN CONCENTRATION BOX PLOT STATISTICS**


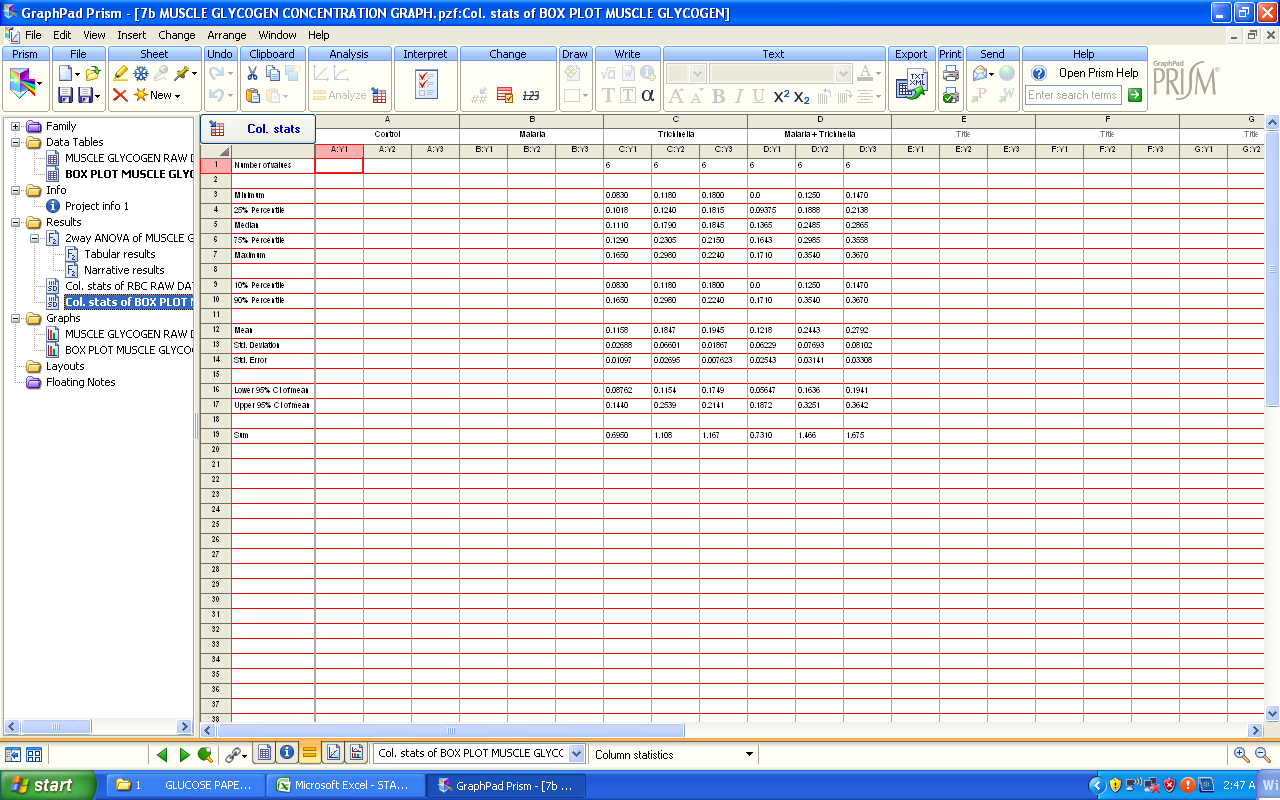

Supplement: Supplemental Information 3 [file peerj-10-13713-s003.docx]
